# The Integrated Care World is a Stage: Applying Goffman’s Theory of Dramaturgy to the Activities of Integrated Care

**DOI:** 10.5334/ijic.8639

**Published:** 2024-08-26

**Authors:** Carolyn Steele Gray, James Shaw, G. Ross Baker, Kerry Kuluski, Walter P. Wodchis

**Affiliations:** 1 Implementing Digital Health Innovation (Tier 2), Canada; 2 Science of Care Institute, Lunenfeld-Tanenbaum Research Institute, Sinai Health, Canada; 3 Institute of Health Policy, Management and Evaluation, Dalla Lana School of Public Health, University of Toronto, Canada; 4 Responsible Health Innovation (Tier 2), Canada; 5 Department of Physical Therapy, Canada; 6 Artificial Intelligence, Ethics & Health, Joint Centre for Bioethics, University of Toronto, Canada; 7 Women’s College Hospital Institute for Health System Solutions and Virtual Care, Canada; 8 Patient and Family-Centered Care, Institute for Better Health, Trillium Health Partners, Canada; 9 Institute of Health Policy, Management and Evaluation, University of Toronto, Canada; 10 Institute of Health Policy Management and Evaluation, University of Toronto, Canada; 11 Implementation and Evaluation Science, Institute for Better Health, Trillium Health Partners, Canada

**Keywords:** integrated care, dramaturgical theory, health system transformation

## Abstract

Among the challenges in delivering integrated health and social care services is the need to attend to the coordination of tasks, roles, activities, and operations, while considering how these efforts are experienced by patients, carers and communities. The literature has noted an important disconnect between how providers and leaders view their efforts to coordinate service delivery, and how patients perceive these efforts on the receiving end. Our team has provided guidance to integrated care efforts in Ontario, Canada by drawing on Goffman’s theory of Dramaturgy to help classify the actions of integrated care delivery as linked to the roles individuals play in the delivery of care. Using this framing helps to uncover how “backstage” processes (such as team-functioning, funding models, and digital infrastructures) create a necessary foundation on which “frontstage” actions (or performances) can be effectively delivered.

## Introduction

The International Foundation for Integrated Care (IFIC)’s 2023 Annual Survey, “Are we there yet?” surveyed IFIC network members (including health care leaders, managers, front line providers, patients and carers, and researchers) about their opinions regarding their regions’ progress towards coordinated and continuous care. Survey findings revealed moderate advancements in continuity and coordination of care delivery globally, with changes remaining largely “embryonic”, local, and varied across countries [1]. A key finding in this survey is that providers may be seeing these advancements, but service users, like patients, carers, and communities, have more negative perceptions of the progress. This discontinuity of experience is a critical challenge, particularly given integrated care’s polymorphous nature, offering different meanings for different actors in the system [2]. This editorial presents Goffmans’s theory of Dramaturgy, a social interactionist theory for understanding human behaviour, as a potentially helpful lens for bridging this divide, with implications for engaging patients, measuring impact, and considering how to effectively advance integrated care.

## Expectations and experiences of integrated care

Integrated care is increasingly characterized as being closely linked to “person-centred care” as evidenced by the World Health Organizations conceptualization of the model [3,4]. From this viewpoint, the experience of ‘person-centredness’ is a key indicator of whether integrated care is achieving its aims. Empirical research has begun to examine what person-centred integrated care entails. For example, Kuluski et al drew on interviews with 172 patients and carers receiving integrated community-based primary care services, identifying six attributes that exemplify high quality integrated care [5]. These six attributes include *easily accessing health and social care that is meaningful, feeling heard, appreciated and comfortable, having someone to count on, knowing how to manage health and what to expect*, and *being independent* while also *feeling safe*. These attributes are rooted in psychological and emotional experiences of care, rather than the number of days it takes to access care or whether diabetes patients hit their target HbA1c levels. Kuluski et al’s findings resonate with other literature on person-centred and goal-oriented care that suggest that meeting the needs of patients and carers requires attending to what is meaningful and valuable in their lives [6].

The value of integrated care for providers and managers also emphasizes person-centredness but sees that as achieved through pursuing greater coordination of activities to improve health outcomes and reducing overall cost [7,8,9,10]. Thus, while providers and patients are both ultimately seeking person-centeredness there is a disconnect in “how” that person-centredness is achieved in models of integrated health and social care. This difference is confirmed in studies exploring the expectations of patients and providers of integrated care [9,10]. For patients integrated care is about having their needs understood, and met, while for providers and systems it is about coordinating activities, emphasizing team composition, infrastructure, leadership approaches and organizational culture [11,12,13]. Systematic reviews suggest the need to make explicit connection between the activities and processes of care delivery by providers and managers and patient experience of care [14,15], which could address this perceived disconnect between what providers and systems are doing, and what patients and carers are actually experiencing.

## Dramaturgy as a helpful metaphor

One way that can help address the divide between patients’ experiences of care, the actions of providers, managers, and the enabling organizational and system environments, is to consider the mechanisms that bridge the gap between action and perception. Erving Goffman’s classic Theory of Dramaturgy (originally presented in 1956) is a sociological perspective which suggests human interaction can be understood as a performance. From this view, actors perform on the “frontstage” for an audience, with the performance being rehearsed and supported through activities and connections occurring on the “backstage,” and sometimes guided by a director who can influence the actions of performers [16]. While the theory was initially intended to help understand human behaviours like impression management (e.g. altering behaviours to be perceived as more socially desirable) in social interactions more broadly, the metaphor can be useful in considering the activities of providers in delivering integrated care services. Dramaturgy has been similarly used to help inform research processes in healthcare [17], understand governance and accountability processes within health systems [18,19], study communication processes and interactions amongst clinicians and providers [20], and explore patient-carer-provider team interactions [21].

Dramaturgy has also been used to help understand organizational processes as “backstage” supports the can influence provider actions and patient experiences. For example, in Ramsey et al’s exploration of patient experiences on the front stage, they define the organizational back stage in terms of scripting (preparatory activities that determine the roles actors are to play), setting (the physical environment in which the play occurs), staging (the deliberate attempts at organizing interactions including the use of props), and performance (referring to the activities that actors are willing to perform) [21]. These backstage activities, which can include processes like dynamic teamwork [22] and supportive technologies [23], are argued to be critical to ensure a “believable performance” (e.g. one that is perceived as genuine by the audience) which is, in turn, important to building trust and demonstrating compassion in care delivery [24]. This example shows how backstage actions can be directly related to the practices that patients witness and experience in care delivery.

Other theoretical lenses stemming from complex systems and realist traditions, such as the Context, Mechanisms and Outcomes of Integrated Care (COMIC) model [25] and realist syntheses on integrated care [26], similarly point to the connections between actions and enabling contexts and mechanisms for integrated care. While these models usefully unpack similar backstage complexities required to enable frontstage activities, they are less explicit on the connection between the behaviours and interactions on the front stage that can influence the perceptions of integration by patients and carers. The dramaturgical view offered by Goffman more closely attends to this interactional and relational nuance which highlights the difference between performance and action on the one hand, and attending to how that action is received on the other; potentially helping those delivering, implementing and studying integrated care to elevate and emphasize how these complexities translate into experiences.

## Connecting performance to backstage activities of providers and managers

In 2019, the authors of this perspective put together a set of guidance documents to support major integrated reform efforts occurring in the province of Ontario in Canada. These guidance documents built on implementation studies conducted by the team as well as the broader literature to help newly forming Ontario Health Teams learn how to put their new models into practice [27]. Dramaturgy was used to scaffold one component of the guideline, connecting front and backstage activities of integrated care (see Box 1 definitions) to the patient experience domains identified by Kuluski [5].

**Box 1 Front and backstage activities of providers and managers defined in the guide [27] (p.9)**

Providers front stage: Patient or carer-facing; any activities involving interactions taking place between providers and patients or their carers, whether synchronous or asynchronous (eg, clinic visits, phone calls, video conferencing, emails).

Providers backstage: Provider or manager-facing; any activities involving interactions with other health or social care providers (internal or external to their organization), volunteers or managers, or independent administrative and preparatory work (eg, charting, case conferencing, training and education) without direct contact with patients or carer.

Managers frontstage: Patient-, carer- or provider-facing; any activities involving interactions with patients, carers, or health or social care providers (internal or external to their organization), volunteers or managers.

Managers backstage: Manager- or partner facing; any activities involving interactions with other managers (internal or external), care delivery partners (from collaborating organizations), policy partners (health ministries and regionally based organizations with a mandate to drive care delivery or quality) and other funders (eg, charitable, not-for-profit and philanthropic funders); or independent administrative and preparatory work (eg, preparing staff meetings, problem resolution, change management, any co-design work).

**Figure 1:**
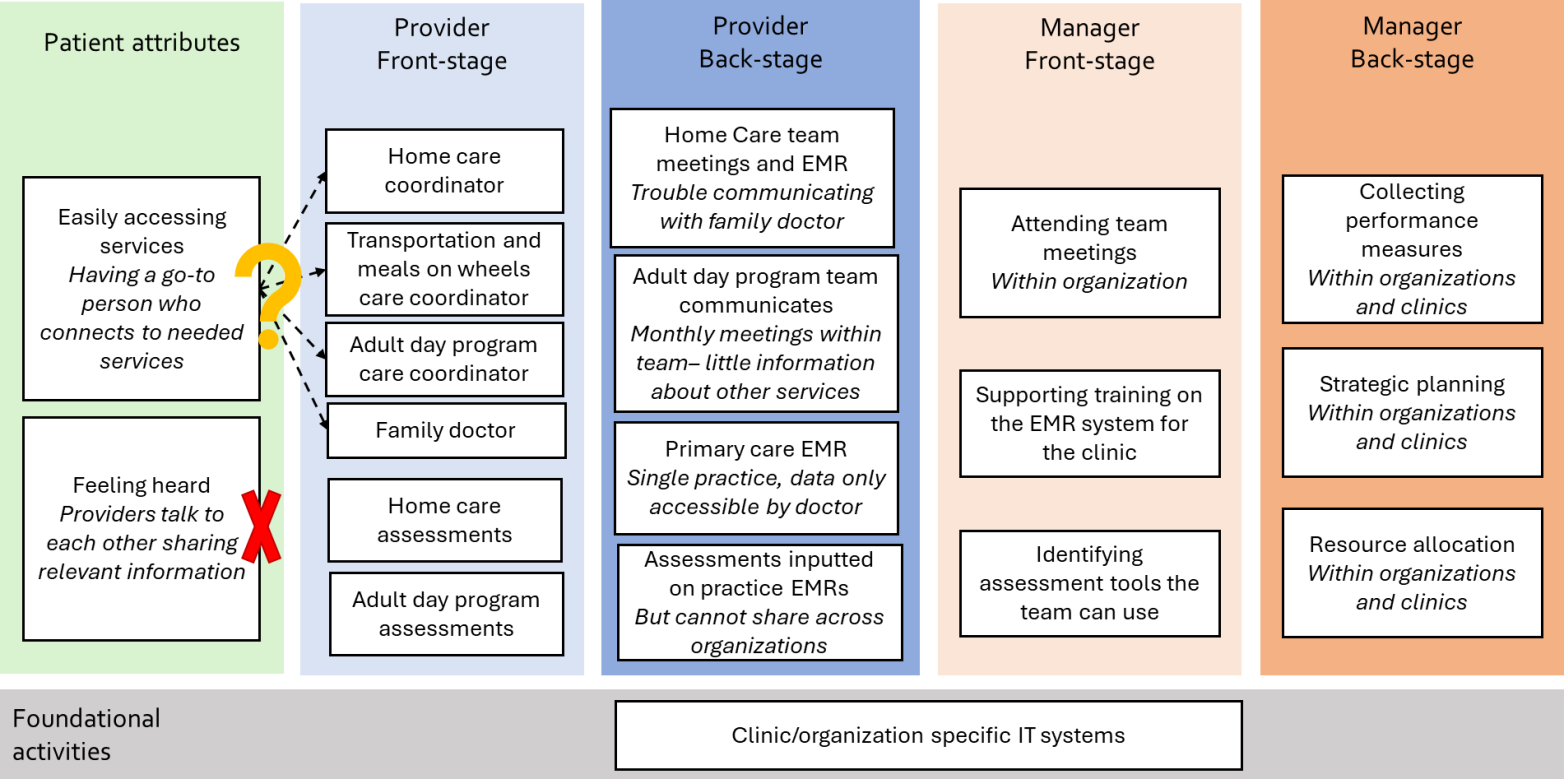
Non-integrated example (modified from Figures presented in [27]). *The? denotes questionable connections between frontstage activities and patient attributes, where the X denotes a lack of connection between frontstage activities and patient attributes.

**Figure 2:**
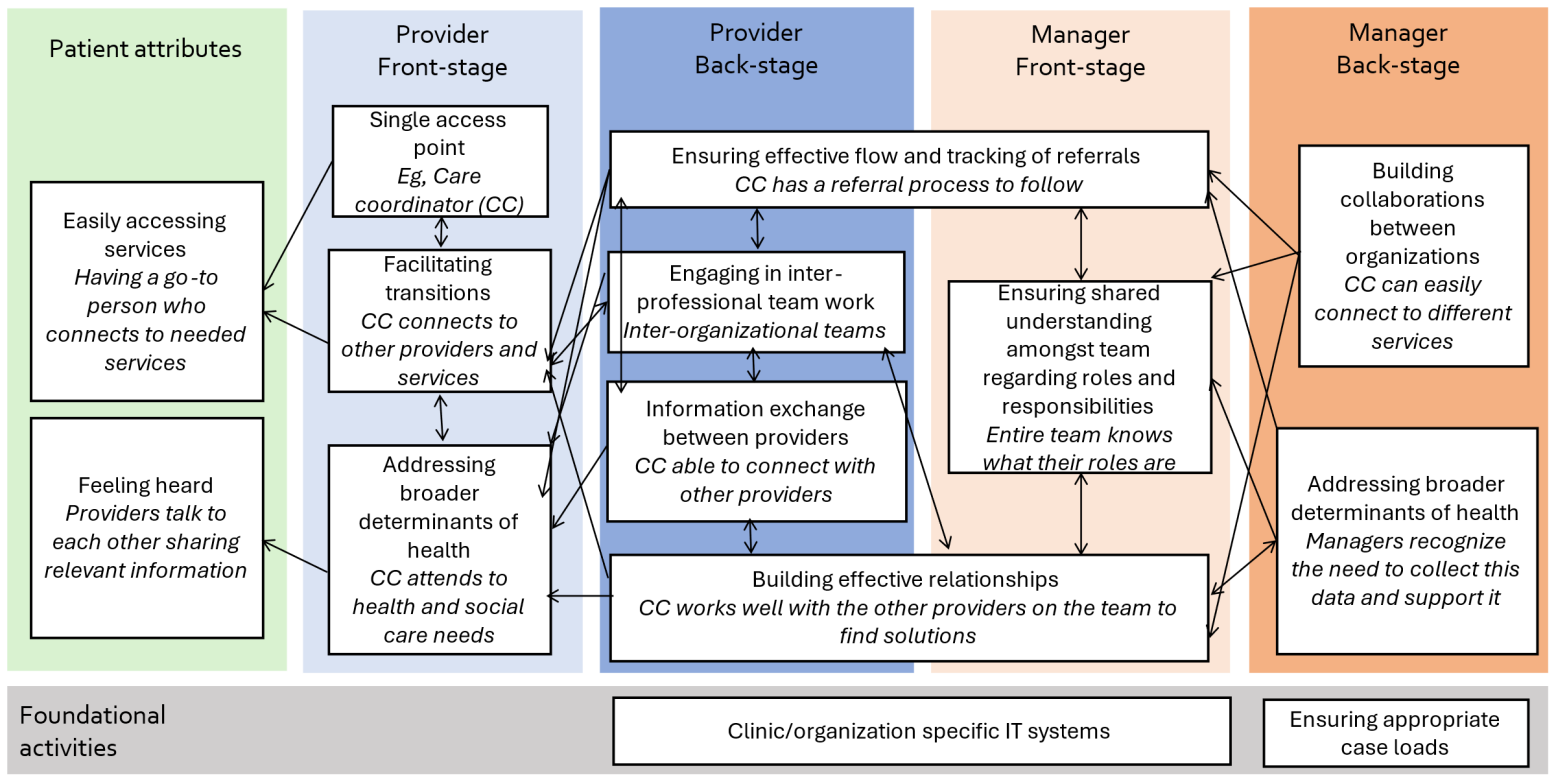
Integrated example.

In the guidance document, 32 front and backstage activities of providers and managers working in integrated care settings were linked to patient experience of care. Figures 1 and 2 included in the guidance document help to visualize the connections between front and backstage activities in non-integrated and integrated examples. The activities listed were derived from empirical research and an exploration of the literature. The metaphor helps to demonstrate the interrelationships of these activities to show teams how they could put processes in place to meet desired experience and outcomes. This approach also helped to illustrate disconnects in the current ways of working that could act as likely barriers to the ability to successfully integrate care.

## How Dramaturgy can help Implementation of Integrated Care

Dramaturgy offers a powerful conceptualization of the relationship between provider behaviours and patient experiences. More practically, it may also offer insights to help implement and improve integrated care. We suggest there are three ways in which the Dramaturgy metaphor can help support implementation of integrated care.

1. ***Supporting patient engagement through critical assessment of roles***. Dramaturgy helps us not only critically assess the performance but also how that performance is being received, meaning that the audience (patients and cares), actors (providers and managers, and where provided sufficient agency to be partners in care, patients and carers as well), and directors (system leaders, institutions, and structures) must all be considered. In this context, it is important to ensure that audience (and actor) feedback can be collected and used to continuously reflect on and adjust as the role of players may need to shift over time, and depending on the scenario. Notably, the role of patients as “audience” may need to be strengthened – more akin to an immersive play experience as seen in Kvæl et al’s work on patient participation in family meetings [21].
2. ***Enabling co-design, co-creation, and co-production of approaches to care***. Mapping the activities and actions of providers and managers delivering care directly to the experience of care by patients and carers, as visualized in Figure 2, can guide co-design and co-production efforts which are becoming increasingly adopted [28]. Making the connection between activity and experience explicit can unearth assumptions about how delivery models work (or don’t) and allow co-design teams to critically assess (and later test) whether these connections hold. This approach is similar to a program theory or logic model approach to understanding the relationship between action and outcome [29,30]. The difference here is in unpacking the connections between structures and activities that may or may not provide an enabling environment for providers to deliver services that are more likely to be experienced as integrated.
3. ***Thinking differently about measuring impact***. We’ve argued that mapping frontstage and back-stage activities to patient and carers experiences of care is akin to program theory and logic model evaluation tools. This approach also encourages us to explore evaluation methods that assess the full pathway, and not just the outcome of our actions. An approach like this aligns to developmental evaluation thinking, and complex evaluation methods [31,32,33], as well as journey mapping and quality improvement and Learning Health System approaches [34,35,36,37]. that encourage us to collect data on processes to iteratively enhance their impact In selecting measures, one or two clear paths can be used as a start to connect the intended outcome (connected to patient and carers experience of integrated care) to the frontstage actions of providers delivering service, and backstage processes and structures that enable them to do their work well. This approach can help untangle some complexity in evaluating these models, allowing for iterative adaptation based on what is learned.

## How to ensure a life-changing performance

Metaphors can be powerful tools for change. They help to make what is implicit more explicit, make the unobserved more obvious, and uncover assumptions that may be influencing our activities and beliefs more than is realized. Here we have argued that Dramaturgy can be a useful metaphor to help bridge the gap between the activities and processes that are put in place to integrate health and social care delivery, with how these services are experienced by the patients and carers. To build on the advancements in integrated care globally, there needs to be deeper understanding of the interconnection between action and experience to guide implementation and evaluation, and, ultimately, to help embed those advancements yielding life-changing integrated care performances.
